# Determinants associated with anemia level among children under 5 years in Gambia: a structural equation modelling approach

**DOI:** 10.3389/fpubh.2025.1598157

**Published:** 2025-07-23

**Authors:** Opeyemi Roselyn Akindutire, Shaun Ramroop, Faustin Habyarimana

**Affiliations:** School of Mathematics, Statistics and Computer, Sciences, University of KwaZulu-Natal, Dubarn, Pietermaritzburg, South Africa

**Keywords:** household income, education, health outcomes, demographic factors, structural equation modelling, anemia

## Abstract

**Introduction:**

Anemia remains a public health concern globally in industrialized and developing nations, especially for children under five. It is still the leading cause of illness and mortality, which is harmful, especially in children from developing countries. Furthermore, children’s emotional, physical, and mental health can be negatively impacted by anemia if it is not addressed promptly. Researchers have unraveled the intricate relationships between components using Structural Equation Modelling (SEM).

**Objective:**

Therefore, using anemia as the main variable, this study intends to use SEM to investigate the association between socioeconomic and household resources and the anemic health status of under-five children in Gambia. The Demographic Health Survey website provided the data for this investigation.

**Methods:**

Twenty-one factors were included in the observations 8,362 utilized in this study and categorized into socioeconomic factors, child’s environmental factors, and household resources. These variables are sex, type of place of residence, marital status, anemia level, type of toilet facility, location of water source, income status, place of residence, month of birth, state, sources of drinking water, and the respondents’ highest level of education. The model specification for the SEM model used shows anemia status level as the dependent variable against income status, parent’s highest educational level, parent’s sex, location of source of water, number of eligible children, state, source of drinking water, parent’s marital status, child’s age and location of toilet facility of the respondents.

**Results:**

The results indicate that parental educational attainment significantly influences children’s hemoglobin levels. The findings indicate that the following factors significantly affect children’s anemia levels: housing location, type of restroom, gender, highest level of education, water source location, marital status, drinking water source, state, number of eligible children, and income status at *p*-value (*p* < 0.000) deliberately.

**Unique contribution:**

The present study has a unique impact of socio-economic factors and human resources on anemia. It would be helpful to consider factors with policy implications for the betterment of anemia patients’ wellbeing.

## 1 Introduction

Anemia among children under five is a significant global public health concern, affecting a substantial portion of the population and remains a major issue worldwide (1). Every year, 20 million infants are born with low birth weight (LBW), and anemia in this group is defined by hemoglobin levels (2). The effects of maternal anemia can extend beyond pregnancy, with newborns potentially suffering from reduced iron stores for up to a year after birth (3).

According to the World Health Organization’s data (4), anemia remains a significant public health challenge affecting children globally, with particularly high prevalence rates in developing countries. The WHO defines anemia in children aged 6–59 months as having a hemoglobin concentration less than 110 g/L, adjusted for altitude, and classifies anemia prevalence of ≥40% in any population group as constituting a severe public health problem requiring immediate intervention.

In The Gambia, anemia persists as a severe public health problem. The 2018 Gambian Micronutrient Survey (GMNS) revealed that approximately 50.4% of children under five were anemic, primarily due to iron deficiency (5). This represents a modest improvement from 2013 levels when approximately 73% of children and 58% of non-pregnant women of reproductive age were anemic (6), but still indicates anemia remains a critical health concern requiring targeted interventions.

Analysis of WHO data from 2000 to 2019 demonstrates that while there has been some progress in reducing anemia prevalence among Gambian children, the reduction rate has been insufficient to meet global nutrition targets. This highlights the need for enhanced, evidence-based interventions that address both the direct nutritional causes and underlying socioeconomic determinants of anemia.

Globally, over one-third of women and more than 40% of children under five suffer from anemia, particularly in rural households with low socioeconomic status and poor sanitation (7, 8). In The Gambia, national data on the nutritional and micronutrient status of women and children was previously limited, with no comprehensive survey that assessed both micro- and macro-nutrient status. The 2018 Gambian Micronutrient Survey (GMNS) aimed to assess the status of iron, vitamin A, and iodine, as well as undernutrition and anemia and its underlying risk factors, to develop new strategies to combat anemia and malnutrition in the country. This paper presents findings from the 2018 GMNS on the nutritional status, prevalence of micronutrient deficiencies, and anemia determinants in children and women (9). Key factors contributing to anemia include nutritional status, where malnutrition and micronutrient deficiencies are crucial determinants (10). A scoping study identified various risk factors for anemia, such as age, sex, maternal education, and household wealth, emphasizing the complexity of the issue (11). While malnutrition encompasses broad dietary inadequacies, a granular understanding of specific nutrient deficiencies is vital for effective interventions. Erythrocyte production, central to preventing anemia, primarily depends on adequate intake of iron, vitamin B12, folate, and protein (49, 54). Protein supply is often directly linked to socioeconomic status and overall income, representing a more indirect pathway to anemia (73). However, micronutrients like iron and vitamin B12 can be addressed through more targeted food-based strategies or supplementation, offering a different intervention avenue (50, 58, 65).

Additionally, community-level factors, like access to healthcare, influence anemia rates among reproductive-age women, indirectly affecting children (12). Addressing these factors through targeted interventions, such as nutritional education and improved healthcare access, is critical for reducing anemia in this vulnerable group (10, 11).

Anemia is a widespread global health issue that affects individuals of all ages. The World Health Organization defines anemia as hemoglobin levels below 12.0 g/dL in women and below 13.0 g/dL in men (13). In pregnant women, anemia is often linked to factors like lack of previous pregnancies, no history of abortions, inter pregnancy intervals shorter than 24 months, and fewer than six prenatal check-ups (14). Among children under 3 years old, risk factors for anemia include diarrhea, being 12 months old, lack of prenatal care, male sex, maternal anemia, and young maternal age (15–24 years) (15). In the old adult, anemia is typically mild (10–12 g/dL) and can result from nutritional deficiencies, chronic diseases, or unknown causes (13). Understanding anemia’s pathophysiology is essential, as it significantly contributes to morbidity and mortality across different populations. The findings of this study indicate that a child’s sex, maternal education level, place of residence, and family size are key determinants of anemia in children under five. These factors were consistently identified as significant contributors to the prevalence of anemia. Therefore, effective interventions should be designed with attention to gender and geographical differences, and address maternal illiteracy through educational initiatives focused on youth and nutrition. Promoting birth spacing is also essential (16).

The connection between socioeconomic factors and anemia in children has gained significant attention in recent years. Research consistently demonstrates that socioeconomic factors, such as household income and educational attainment, are crucial in influencing health outcomes. Higher household income and better educational levels are often associated with improved health outcomes, including reduced rates of chronic illnesses, lower mortality, and overall better wellbeing. Studies have shown that household income can directly affect health by enhancing access to essential resources, such as safe housing, nutritious food, and healthcare. Education, in contrast, may influence health indirectly through factors such as health literacy, lifestyle choices, employment opportunities, and social support networks.

Understanding the complex interplay between socioeconomic factors, household resources, and anemia is essential for developing effective public health strategies. Structural Equation Modelling (SEM) provides a robust methodological approach to examine these multifaceted relationships, allowing for the identification of both direct and indirect pathways through which socioeconomic and household factors influence anemia status in children under 5 years in The Gambia.

Addressing the important issue of anemia following research questions are raised below.

### 1.1 Research questions

*RQ1*: What are the factors affecting the anemia level of a person?

*RQ2*: What are the factors affecting the household resources of an anemia person?

*RQ3*: What is the effect on various socio-economic aspects of anemic person?

### 1.2 Research hypothesis

*H01*: There is no significant effect of factors on anemia level.

*H02*: there is no significant effect of Household resources on anemic person.

*H03*: there is no significant effect of socio-economic aspect on anemia patients.

Anemia affects over one-third of women and 40% of children under five globally, disproportionately impacting rural households with low socioeconomic status and poor sanitation (7, 8). In The Gambia, national data on the nutritional and micronutrient status of women and children is outdated, with no comprehensive survey that assesses both micro- and macro-nutrient status. In 2013, approximately 73% of children and 58% of non-pregnant women of reproductive age were anemic (Gambia, 2014), though key anemia risk factors like iron deficiency, malaria, and inflammation had not been measured. The 2018 Gambian Micronutrient Survey (GMNS) aimed to assess the status of iron, vitamin A, and iodine, as well as under nutrition and anemia and its underlying risk factors, to develop new strategies to combat anemia and malnutrition in the country. There are inadequate studies on the impact of socioeconomic factors and human resources on anemia level. The consideration of factors on policy implications is necessary to improve the health status of anemia patients.

To address the identified research gap, it is essential to design appropriate interventions that consider differences in sex and residence. These interventions should focus on improving maternal education, particularly through youth and nutrition education, and promote birth spacing (16). Additionally, strengthening the free maternal health insurance scheme and encouraging registration for women without health insurance is necessary, as maternal health insurance has been shown to reduce the likelihood of anemia in children. Policies aimed at providing sufficient maternal leave for employed nursing mothers should also be pursued, as children of employed mothers are more likely to experience fever compared to those of unemployed mothers. Alternatively, establishing better-regulated childcare facilities could offer employed mothers the option to ensure proper care for their children when their work schedule does not allow sufficient time for caregiving. Furthermore, long-term poverty reduction strategies should be implemented, along with supporting low-income households in adopting better child health practices, as children from wealthier households are less likely to experience diarrhea, fever, or anemia compared to those from poorer households (17). It is also important to note that previous studies did not account for biological factors, food security, or dietary influences on anemia, nor did they include other relevant socioeconomic factors such as economic activity, social class, or income. Additionally, the cross-sectional nature of the data prevented the establishment of temporality or causality. Given that the study was quantitative, qualitative research is needed to explore mothers’ and caregivers’ perspectives on the low enrollment in the National Health Insurance Scheme (18).

The theoretical foundation for this study is primarily based on Grossman's (19) work, which posits that individuals are active producers of their health and that various factors contribute as inputs into the health production function. This model emphasizes the influence of factors such as age, education, and income on health outcomes. However, to better understand the socio-economic factors affecting child health, the model developed by Mosley and Chen (20) is particularly relevant. In related research, Escobar et al. (21) in Brazil found that younger children, malnourished, had mothers with low education, belonged to lower-income households, or lived with another child suffering from diarrhea were at higher risk of contracting the disease. In Ghana, Quansah, Ohene, Norman, Mireku, and Karikari (22) identified key determinants of child health, such as family income, maternal education, place of residence, and high dependency (multiparity) through a qualitative study of existing literature.

Between 2020 and 2024, several studies have employed Structural Equation Modelling (SEM) to explore the direct and indirect effects of household income and education on health outcomes. These studies have deepened the understanding of the relationship between socioeconomic status and health, highlighting the significance of both income and education, individually and together. This study, therefore, aims to examine the relationship between income status, the highest level of education, and health status, focusing on anemia as the key health indicator, using SEM. The structure of this paper is as follows: The Abstract is presented first, followed by the Introduction, which incorporates the literature review. Section 3 discusses the materials and models used, Section 4 presents the results, Section 5 provides the discussion, and Section 6 concludes the paper.

Anemia has significant implications for both human health and socioeconomic development. In children, it is linked to impaired mental and physical development, along with higher rates of morbidity and mortality (23). Socioeconomic factors such as maternal education and access to clean water have notable independent effects on hemoglobin levels. In addition to iron deficiency, factors such as maternal hemoglobin levels, family wealth, the child’s age and gender, were identified as contributing to anemia among young children in rural India (24). This research found that maternal education is a key predictor of anemia in children under five. Children of mothers with no formal education were 53% more likely to suffer from anemia (25). This is likely because mothers with little education may lack awareness of proper feeding practices and are less likely to follow nutrition guidelines (26). Furthermore, uneducated mothers often face lower socioeconomic status, which limits their ability to purchase nutritious food, thus exacerbating the risk of nutritional deficiencies in their children. Therefore, nutrition education for mothers is recommended to reduce anemia in children (27).

Research by Amugsi et al. (28) highlights maternal weight, number of young children, residence, wealth index, and the mother’s age in predicting children’s growth outcomes in Ghana. Consistent with previous studies (29–31), this study found that children from impoverished households are at a higher risk of anemia than those from wealthier families. Numerous studies have demonstrated the link between socioeconomic status and health outcomes. Higher wealth and educational attainment are associated with longer life expectancy, better mental health, and lower rates of chronic disease. Research also shows that children’s cognitive and social development positively correlates with household income and overall health status. A study revealed that family size was a significant factor in the risk of anemia among children under five in Africa. Children from households with fewer than five members were 7% less likely to be anemic compared to those in larger families. This may be because smaller families tend to experience less food insecurity, allowing for a more adequate and diverse diet, rich in iron (32).

The causes of anemia in children under five are complex, with contributors including low birth weight, malnutrition, poor socioeconomic status, food insecurity, inadequate breastfeeding duration, poor dietary iron intake, low maternal education, illness, poverty, inadequate sanitation, and monotonous diets (24, 33, 34). Diggle (35) found that the absence of toilet facilities was associated with higher risks of malnutrition in children, in addition to factors like multiple births, poverty, extended breastfeeding periods, and a history of diarrhea. This study found that residence type (rural vs. urban) was a significant factor in anemia risk among young children. Children living in rural areas were 20% less likely to suffer from anemia compared to those in urban settings. This could be due to mothers in rural areas more likely to exclusively breastfeed their children for the first 6 months and continue breastfeeding beyond that, which ensures better iron absorption and reduces the risk of anemia. UNICEF and WHO recommend adequate breastfeeding practices as a key strategy to combat anemia in children (25, 27). In The Gambia, anemia remains a major public health issue, affecting a significant percentage of both children (50.4%) and reproductive-age women (44.28–50.9%) (5, 12). Iron deficiency is a major contributor, with 38.2% of children experiencing iron deficiency anemia (5). Women’s empowerment, including maternal education and attitudes toward domestic violence, plays a critical role in improving child nutrition and reducing stunting and underweight conditions (36). Factors such as pregnancy, contraceptive use, and proximity to health facilities are also associated with anemia risk in women (12). Research from Ghana suggests that health insurance may help reduce anemia among children under five (18), emphasizing the need for comprehensive strategies addressing both nutritional and socioeconomic factors. The importance of considering socioeconomic factors in health outcomes is clear from existing research. Studies consistently show that higher wealth and education are correlated with better health outcomes, including longer life expectancy, better mental health, and reduced chronic diseases. These findings underline the need for policies that address the multifaceted impact of socioeconomic status on public health. The type of cooking fuel used is associated with air quality, particularly indoor air pollution. Studies suggest that solid fuels (e.g., wood, coal, and biomass) often lead to indoor air pollution, which can contribute to respiratory and cardiovascular problems, and indirectly affect nutritional status, leading to anemia. Long-term exposure to smoke from these fuels may exacerbate conditions like iron deficiency anemia by influencing nutrition and absorption. Studies have shown that in areas where cleaner fuels (e.g., electricity, gas) are used, the incidence of anemia tends to be lower. This is likely due to the indirect effects of improved air quality, which may allow for better absorption of nutrients and healthier environments overall. The use of the Internet can be an indicator of education, awareness, and access to healthcare information, which can indirectly influence nutritional habits and health management. For example, access to online health resources may increase awareness about iron-rich foods, dietary practices, and anemia prevention. While the direct connection between internet use and anemia is less studied, increased digital literacy could correlate with better health practices, potentially reducing the prevalence of anemia, especially if individuals can access better dietary information. Owning a mobile phone can be useful for socio-economic status. Individuals with mobile phones may have better access to health services (through telemedicine) and information, potentially improving their health literacy. While there is no direct link to anemia, socio-economic status is a known determinant of health outcomes, including anemia. In regions where healthcare infrastructure is weak; mobile phone ownership facilitates communication and access to health advice, which can indirectly help manage conditions like anemia. The material used for the roof of a home can indicate the overall socio-economic status of the household, which impacts access to resources such as nutrition and healthcare. Poor housing conditions may lead to higher vulnerability to infections and poor nutrition, both of which are risk factors for anemia. Homes with poor-quality roofs (e.g., thatch or corrugated metal) may have higher rates of anemia due to their association with lower-income families, poorer sanitation, and malnutrition, which are contributing factors. Like roof material, the type of wall material can indicate the quality of housing, which affects living conditions and overall health. Poor housing quality is often linked to lower access to clean water, sanitation, and balanced diet factors that increase the risk of nutritional deficiencies, including anemia. Homes with walls made of mud or poorly insulated materials might correlate with poor ventilation and higher exposure to infections, both of which can impact nutrition and anemia risk. The material of the floor may also correlate with living conditions and hygiene, particularly concerning soil-transmitted helminthes (worms) and other infections that cause malnutrition and anemia. Poor-quality floors may lead to a higher risk of infections that, in turn, can worsen iron deficiency. Floor materials like dirt or low-quality concrete might correlate with poor hygiene and increase exposure to parasites, which can exacerbate anemia through blood loss or malnutrition. The presence of electricity in the household is a strong indicator of socio-economic status. Households with electricity have greater access to refrigeration, cooking appliances, and healthcare, all of which can contribute to better nutrition and health outcomes, reducing the risk of anemia. Studies indicate that electrified homes have a lower incidence of anemia, as electricity enables the use of modern cooking methods, refrigeration (preserving iron-rich foods), and better access to health services. In contrast, households without electricity often depend on traditional cooking methods, which can have detrimental effects on health.

Ownership of a television can be linked to better access to information, including health-related content. This may correlate with higher health literacy and awareness of anemia, dietary changes, and iron supplementation, which can reduce anemia prevalence. While not directly related to anemia, owning a television is often associated with increased awareness of public health campaigns regarding anemia prevention, iron supplementation, and nutrition. A refrigerator allows households to store perishable foods, including iron-rich items like meats and dairy products, which can improve nutritional intake. Households without refrigerators may be more reliant on non-perishable, often iron-deficient foods, which can exacerbate anemia. Studies show that households with refrigerators tend to have better food security and nutrition, which can directly reduce the prevalence of anemia by facilitating a diet rich in iron and other micronutrients.

In addition, Malaria, which has fever as one of its major signs, is a public health problem in Ghana with children being one of the most vulnerable groups. Also, Pneumonia, which has cough as some of its symptoms combined with some respiratory tract infections, is a major killer of younger children in Ghana (37). Moreover, it has been reported that the death of children in Ghana that can be attributed to diarrhea and pneumonia significantly increased in the 2012–2013 period (38). Also, the presence of the anemia control program in Ghana, tells us about the significance of tackling anemia to the health of the entire population in Ghana, which includes child health. Therefore, it was not surprising that the GSS, GHS, and ICF International (37) contend that 66% of children in Ghana are anemic (17).

This study showed that sex was a significant predictor of anemia among children. Being female is protective against anemia among children. %e possible explanation for anemia discrepancy by sex could be due to the state of rapid growth of male children compared to females in the first months of life, which increases their micronutrient requirement, including iron, which cannot be met by diet alone (39). If this physiological state is not compensated with iron-rich complementary foods, the risk of iron deficiency anemia will be higher in male children as compared to females.

However, the literature on health insurance and child health outcomes is mixed. For example, studies conducted in China and Costa Rica found no significant relationship between health insurance membership and child health outcomes (40, 41). On the contrary, Nshakira-Rukundo (66) found that household enrolment in health insurance was significantly associated with a 5.7% reduction in child stunting. In addition, studies conducted in the United States of America found that the state children’s health insurance program brought improvement in child health outcomes (42, 43). Furthermore, evidence from Taiwan shows that health insurance reduces the incidence of death among children (55). Nationally, wasting, underweight, and stunting, denote only mild to moderate public health problems in Gambian children. Furthermore, children whose mothers had health insurance were revealed to be 4% less likely to suffer from anemia relative to those with uninsured mothers. This is in line with the result of Aheto et al. (44) who revealed that the non-existence of maternal health insurance is linked with a higher risk of malnutrition. The reason is that health insurance makes medical care relatively cheaper, and hence mothers would utilize it for themselves and their children, especially in the case of the national health insurance scheme in Ghana, where there is free health insurance registration for children and pregnant women. Also, since mothers with health insurance are more likely to visit health facilities, they may get health education on good child health practices as compared to their uninsured counterparts. This recommendation is in line with previous scholarly work that, along with documenting an association between malaria and anemia (45), has shown that malaria prevention efforts, such as indoor residual spraying (46) and the use of insecticide-treated nets (77), is associated with lower rates of anemia. Researching the relationship between various household and demographic factors (such as type of cooking fuel, access to technology, and material types in housing) and anemia involves exploring how socio-economic, environmental, and lifestyle factors influence the prevalence of anemia.

SEM is an effective statistical method that allows scientists to examine several correlations between variables simultaneously. It also highlights positive effects on intermediate outcomes such as maternal mental health and parenting quality. This can be linked to the economically poor population’s greater levels of stress, unhygienic living conditions, and restricted access to healthcare services. Research by Godha et al. (60) and Gressel et al. (57)emphasizes the significant role of education in determining health outcomes. Higher levels of education are often linked to better health behaviors, increased access to healthcare, and improved socioeconomic status, all of which contribute to overall better health outcomes. The interaction between income and education on health outcomes is complex. Some studies, such as those by Thaddeus and Maine (74), suggest that the health benefits associated with higher education may be more pronounced among individuals from lower-income households, indicating a potential buffering effect of education on the adverse health effects of low income. They all agree that: understanding the relationship between household income, education, and health outcomes is crucial for informing public health policies aimed at reducing health disparities. Interventions targeting education access, income inequality, and healthcare accessibility can play a significant role in improving overall population health.

These findings underscore the importance of addressing socioeconomic determinants such as income and education to achieve health equity and improve the wellbeing of communities. According to previous study, many possible indicators could indicate how complex the relationship is between health and education. These indicators include but are not limited to, the interrelationships between family background and demographic indicators, the effects of childhood illness, the greater resources that come with higher education levels, the value of education, and more. Research indicates that educational attainment is positively associated with better health outcomes, including higher self-reported health, lower morbidity, and lower mortality (47, 69). Some statistics indicate that there is a significant relationship between education and health determinants, particularly preventative care. Education strengthens bonds between people, encourages the upkeep of sensible choices and healthy lifestyles, and enhances the wellbeing of the individual, the family, and the community (63). However, there are drawbacks to schooling as well. Education may result in a stronger emphasis on preventative care, which raises healthcare costs temporarily despite its long-term advantages. Research has found a connection between education and the use of several illicit drugs and alcohol. In conclusion, studies have indicated that although education is considered beneficial in treating depression, its impact on general happiness or wellbeing is far smaller (70).

The Johansen co-integration test indicates that income and household consumption expenditure have a long-term relationship. Using the error correction model, the mistakes that surfaced in the short run were fixed in the long run. Income and household consumption expenditures have a strong and direct link, and this relationship is also present for all other variables apart from inflation. An indirect relationship can be seen in inflation. Given that household consumption expenditure and income were found to be positively correlated with time, the study suggested that the government step up its welfare initiatives to increase the purchasing power of its inhabitants. To entice current and new investors, the interest rate ought to be lowered. The monetary authorities ought to implement measures that guarantee both price stability and a decline in inflation rates. As a result, each household’s income would increase in value, raising household consumption (64). A study by Owolabi et al. (68) found that individuals with high levels of education were more likely to engage in healthy behaviors such as exercise and healthy eating, leading to better health outcomes. The SEM model also suggests that income and education have significant direct effects on health outcomes, even after controlling for access to healthcare, lifestyle choices, and social determinants of health. Furthermore, the model reveals several indirect pathways through which income and education influence health outcomes. For example, higher income levels are associated with better access to healthcare, which in turn leads to improved health outcomes. Similarly, education is linked to healthier lifestyle choices and greater social support, which contribute to better health outcomes.

## 2 Materials and methods

### 2.1 Data for the Study

The data used for this study is secondary data obtained from the Demographic Health Survey. After cleaning and removing missing observations, 8,362 observations were used for this study. The 21 variables extracted for this study are sex, type of place residence, marital status, anemia level, type of toilet facility, location of source for water, state, number of eligible children, source of drinking water, location of type of toilet facility, income Status, and the highest educational level of the respondents.

#### 2.1.1 Participant selection criteria

Children aged 6–59 months with complete hemoglobin measurements were included in the analysis. The study focused specifically on children under 5 years in The Gambia with valid anemia status data. Anemia was classified according to WHO standards as follows: severe anemia (<70 g/L), moderate anemia (70–99 g/L), mild anemia (100–109 g/L), and not anemic (≥110 g/L). These thresholds were adjusted for altitude as recommended by WHO guidelines.

Records with missing values for key variables, including hemoglobin level, parental education, type of place of residence, and other critical socioeconomic indicators (*n* = 17,629) were excluded from the analysis. Additionally, invalid or biologically implausible hemoglobin values (*n* = 432) were removed to ensure data quality. The final analytical sample consisted of *N* = 8,362 observations with complete data on all variables of interest.

### 2.2 Structural equation modelling

Structural Equation Modelling (SEM) is a multivariate statistical technique that has been investigated to assess correlations between variables (68). A limitation of traditional statistical approaches, such as path analysis and regression analysis is their assumption that variables are measured without error. SEM addresses this limitation by explicitly modelling measurement error in the relationships between variables. As a result, this research clarifies the definition of SEM as well as its underlying presumptions, procedures, and some terminology. A brief analysis of item parceling’s significance to SEM and its techniques was conducted. It also covered the steps in SEM, the parallels and discrepancies between SEM and traditional statistical techniques, and software tools for SEM. Additionally, SEM is helpful in reducing measurement errors and improving construct reliability. Considering this, it is advised that SEM be used to examine complicated interactions between variables, including spurious, direct, indirect, hierarchical, and non-hierarchical associations. SEM clearly distinguishes between latent and manifest variables and enables the assessment of model fitness using a variety of indices, including comparative fit, absolute fit, residual-based fit, and the percentage of variation accounted for indices. Since SEM may examine intricate links between variables, including direct, indirect, spurious, hierarchical, and non-hierarchical relationships; it should be used to test interactions between variables.

A thorough statistical method for assessing and estimating causal links that combine quantitative data with qualitative causal hypotheses is structural equation modelling or SEM. The social sciences, behavioral sciences, and economics can all benefit greatly from SEM since it makes it possible to analyze intricate correlations between latent and observable variables (53).

#### 2.2.1 Confirmatory factor analysis

The measuring part of the model is the focus of this approach. It analyses how well the observed variables represent the latent variables and uses several observed variables (indicators) to identify underlying latent variables (unobservable constructs). It determines if your hypothesized latent constructs, or unobservable characteristics, are reflected in a set of observed variables. CFA is used to test the measurement model, which specifies the relationships between observed variables and their underlying latent constructs (see Equation 1).

Equation for CFA:


(1) $$Y=\Lambda_{y}\;\eta +\in$$

Where:

Y represents the observed variables, Λ is the matrix of factor loadings, η denotes latent variables, and ϵ is the measurement error.

#### 2.2.2 Path analysis

Path analysis examines relationships among observed variables only, without latent constructs. Unlike confirmatory factor analysis, which focuses on measurement models with latent variables, path analysis tests direct and indirect effects between measured variables using a series of regression equations represented in path diagrams. It is considered a precursor to full structural equation modelling that allows for the examination of causal relationships among a set of observed variables.

The path model (Equation 2) can be expressed as:


(2) $$\eta_{i}=\beta_{\mathrm{i0}}+\sum \beta_{\mathrm{ij}}\eta_{j}+\varsigma_{i}$$

Where ηi is the Score on latent variable I, β_i0_ is the intercept for latent variable I, β_ij_ the path coefficient from latent variable j to latent variable i (represents the strength and direction of the influence), $\eta_{j}$ is the score on the latent variable j and ζ_i_: Error term for latent variable i (represents unexplained variance).

#### 2.2.3 Structural model equation

The structural model describes the relationships between endogenous and exogenous latent variables. It combines the measurement and path analysis models to provide a comprehensive view of the hypothesized relationships as shown in Equation (3).


(3) η=Bη+Γξ+ς

Where:

η is the vector of endogenous latent variables, B is the matrix of coefficients relating endogenous variables to each other, Γ is the matrix of coefficients relating exogenous variables (ξ) to endogenous variables, and ζ represents the vector of structural disturbances.

### 2.3 Study model specification for the anemia status

**Figure 1:**
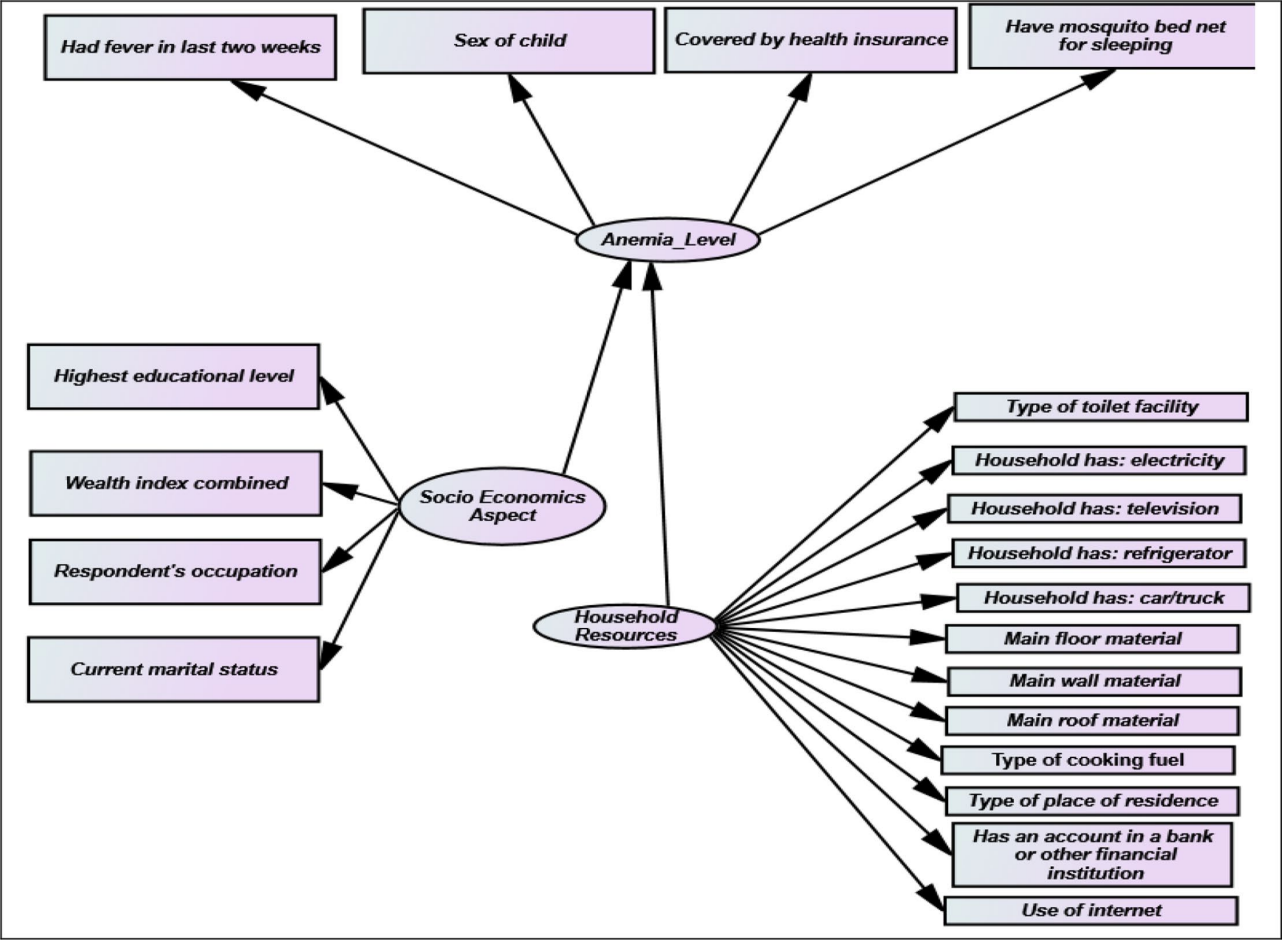
Feature variables for the study.

This section presents the specification of the Structural Equation Model used in this study. Anemia status level is considered as the dependent variable against income Status, highest educational level, sex, month of birth, number of eligible children, place of residence, marital, type of toilet facility, state, and location of toilet facility of the respondents as shown in Figure 1. The second model shows the income status as the dependent variable against the type of place of residence and the highest educational level of the respondents. The Chi-square test is used to show a direct relationship between the dependent variables and the outcome variable.

This analytical method is a two-step procedure. The first phase involves testing putative measures of the underlying constructions with confirmatory factor analysis. The process results in the development of a measurement model wherein the degree of departure of an item belonging to a latent variable from the standardized regression estimate B value of the indicator variable’s path to the latent variable inside the model serves as an indicator of its validity. These indicators include average variance extracted, composite reliability for overall reliability, and squared standardized loading for individual item dependability. The path significance or standardized regression estimates are the same criteria used for evaluating the measurement model and the structural model. Fit index is also used in the evaluation of the measurement and structural models. The hypothesis states that gender, place of residence, type of toilet facility, the highest level of education, income status, state, number of eligible children, month of birth, source of drinking water, location of toilet facility, and the location of the source of water have a significant effect on the anemia level of the patients. The statistical analysis was performed using R software (version 4.2.1) with the lavaan package (version 0.6–11) for structural equation modelling. Additional packages included *semPlot* (version 1.1.3) for model visualization, psych (version 2.2.5) for reliability analysis, and *corrplot* (version 0.92) for correlation matrix visualization.

## 3 Results

This section presents and discusses the primary empirical findings of the investigation. The frequency distribution of the demographic variables is shown in Tables 1, 2, and the reported estimates of the SEM system are shown in Table 3. The estimates for the Anemia status and disability measuring equations are shown in the second column, and they are all significant. Several closing observations can be drawn from the table.

**Table 1:** Demographic and socioeconomic characteristics of study participants (*N* = 8,362).

Variable	Categories	N	%
Anemia level	Severe	148	1.80%
	Moderate	996	11.90%
	Mild	1,159	13.90%
	Not anemic	2,042	24.40%
	Missing (−99)	4,017	48.00%
Has mosquito bed net	No	594	7.10%
	Yes	7,768	92.90%
Covered by health insurance	No	8,212	98.20%
	Yes	150	1.80%
Sex of child	Male	4,364	52.20%
	Female	3,998	47.80%
Had fever in last 2 weeks	No	6,444	77.10%
	Yes	1,326	15.90%
	Do not know	157	1.90%
	Missing (System)	435	5.20%
Highest education level	No education	4,439	53.10%
	Primary	1,555	18.60%
	Secondary	2,148	25.70%
	Higher	220	2.60%
Wealth index	Poorest	2,949	35.30%
	Poorer	1,808	21.60%
	Middle	1,612	19.30%
	Richer	1,141	13.60%
	Richest	852	10.20%
Current marital status	Never in union	227	2.70%
	Married	7,910	94.60%
	Living with partner	8	0.10%
	Widowed	72	0.90%
	Divorced	126	1.50%
	Separated	19	0.20%
Respondent’s occupation	Not working (last 12 months)	2,537	30.30%
	Managers	14	0.20%
	Professionals	113	1.40%
	Technicians/associate professionals	55	0.70%
	Clerical support workers	43	0.50%
	Service/sales workers	2,301	27.50%
	Skilled agricultural/forestry/fishery workers	2,861	34.20%
	Craft/trades workers	205	2.50%
	Plant/machine operators	13	0.20%
	Elementary occupations	203	2.40%
	Other	16	0.20%
Type of residence	Urban	3,749	44.80%
	Rural	4,613	55.20%
Toilet facility	Flush to sewer/septic/pit	1,797	21.50%
	Pit latrine (with/without slab)	5,836	69.80%
	No facility/bush/field	164	2.00%
	Other/Not dejure resident	575	6.90%
Household assets	Electricity (Yes)	3,943	47.20%
	Television (Yes)	4,236	50.70%
	Refrigerator (Yes)	2,862	34.20%
	Motorcycle/scooter (Yes)	2,443	29.20%
	Car/truck (Yes)	1,362	16.30%
Housing materials	Cement/concrete floor	3,647	43.60%
	Cement walls	6,267	75.00%
	Metal roof	6,884	82.30%
Cooking fuel	Wood	6,193	74.10%
	Charcoal	1,702	20.40%
	Other	471	5.60%
Financial access	Owns mobile phone (Yes)	5,867	70.20%
	Has bank account (Yes)	848	10.10%
Internet use	Never	3,802	45.50%
	Used in last 12 months	4,187	50.10%
	Used before last 12 months	373	4.50%

**Table 2:** Reliability statistics.

Cronbach’s alpha	N of items	Sample size N
0.837	22	8,362

Source: Authors calculation.

**Table 3:** Reliability, B, SE, CR *P*-values.

DV	IV	Reliability	B	SE	CR	*P*
Anemia level	Had fever in last 2 weeks	0.839	1			
	Sex of child	0.839	−0.562	0.681	−0.824	0.41
	Covered by health insurance	0.839	0.97	0.933	1.04	0.298
	Have mosquito bed net for sleeping	0.839	−4.621	4.42	−1.045	0.296
Household resources	Household has: electricity	0.832	1			
	Household has: television	0.832	0.994	0.003	287.36	***
	Household has: refrigerator	0.832	1.014	0.003	317.583	***
	Type of toilet facility	0.798	10.898	0.072	151.538	***
	Type of place of residence	0.839	−0.046	0.004	−11.08	***
	Type of cooking fuel	0.794	13.138	0.062	211.609	***
	Use of internet	0.838	0.043	0.005	8.799	***
	Owns a mobile telephone	0.839	0.026	0.004	6.529	***
	Has an account in a bank or other financial institution	0.839	0.012	0.003	4.621	***
	Main roof material	0.792	10.3	0.055	188.234	***
	Main wall material	0.792	10.179	0.053	193.688	***
	Main floor material	0.798	10.302	0.07	147.439	***
	Household has: car/truck	0.832	1.029	0.005	225.774	***
Socio economics aspect	Highest educational level	0.838	1			
	Current marital status	0.839	−0.046	0.014	−3.305	***
	Wealth index combined	0.839	3.028	0.278	10.893	***
	Respondent’s occupation	0.843	−0.275	0.138	−1.991	0.046

Source: Author’s calculation.

***indicates statistical significance at *p* < 0.001 level, meaning the corresponding *p*-value is less than 0.001. This denotes a very strong level of statistical significance, as the findings in those rows are highly statistically significant.

Table 1 presents the frequency distribution of the study’s variables. The results analyze several socio-demographic and health-related factors among respondents. Anemia levels show that 1.8% of the respondents were categorized as severe anemia, 11.9% had moderate anemia, and 13.9% had mild anemia. About 24.4% were not anemic; 48% of the information on anemia status was absent. Regarding preventive health practices, 92.9% of homes said they slept on a mosquito bed net; 7.1% did not. Furthermore, just 1.8% of the answers were insured, hence 98.2% lacked health care. Child respondents’ sex distribution was quite even, with 52.2% male and 47.8% female participants. Data on fever incidence showed that 15.9% said they had had a fever in the recent 2 weeks; 77.1% said they had not. Of the replies, 5.2% were lacking and 1.9% said “Do not know.” Of the 53.1% who had no formal education, 18.6% had finished primary school, 25.7% had secondary education, and just 2.6% had higher education. Regarding wealth position, 35.3% of those polled were deemed the poorest, 21.6% poorer, 19.3% middle class, 13.6% richer, and 10.2% the richest. Data on marital status revealed that most (94.6%) were married, 2.7% had never been in a partnership, and 0.1% were cohabiting with a partner. Of the rest, 0.9% were widowed, 1.5% divorced, and 0.2% separated. According to occupational statistics, 34.2% of respondents worked in skilled agriculture, forestry, and fisheries jobs; 30.3% were not working or had not worked in the last 12 months; and 27.5% were involved in service and sales activities. Of the respondents, 55.2% lived in rural areas and 44.8% in urban. Regarding cleanliness, 40.8% of those surveyed said they used a pit latrine without a slab or open pit; 29% said they used a pit latrine with a slab; and 12.9% said they had a flush toilet linked to a septic tank. Data on asset ownership showed that 34.2% of homes had a refrigerator, 50.7% had a television, and 47.2% had power. Of the others, just 16.3% had a truck or car. Of those polled, 70.2% had a mobile phone; just 10.1% had a bank account. While 45.5% had never used the internet, 50.1% had used it in the last 12 months. With respect to house materials, the main floor material stated was cement or concrete (43.6%), followed by tiles (19.6%) and vinyl/linoleum (18.0%). While 8.7% were thatched with palm leaf, the rest of roofs (82.3%) were metal corrugate. Cement (57.8%) was the most reported main wall material; 16.0% used mud or mud bricks, 9.6% used mud blocks covered with cement. Cooking fuel-wise, 74.1% of homes used wood, 20.4% used charcoal, and 0.6% used LPG. Of those polled, 29.8% lacked a cell phone and 89.9% lacked a bank account, suggesting poor financial resource availability. The data overall shows notable differences in socioeconomic status, health indicators, and access to necessary services among the respondents; rural people seem to be more disadvantaged in terms of asset ownership, educational level, and health insurance coverage (53).

Conbach’s alpha value for the confirmatory factor analysis is 0.837. Cronbach’s alpha can be directly interpreted; a score of.70 or higher is typically regarded as satisfactory. High consistency is defined as 0.90 or higher, good consistency as 0.80–0.89, acceptable consistency as 0.70–0.79, marginal consistency as 0.50–0.69, and unacceptable consistency as less than 0.5. Cronbach’s alpha score for each item is lying in between 0.792–0.843, so that is highly recommended for further analysis. Alpha scores between 0.7 and 0.8 are considered adequate for scales that are utilized as research tools to compare groups (75).

### 3.1 Confirmatory factor analysis

#### 3.1.1 CFA model

**Figure 2:**
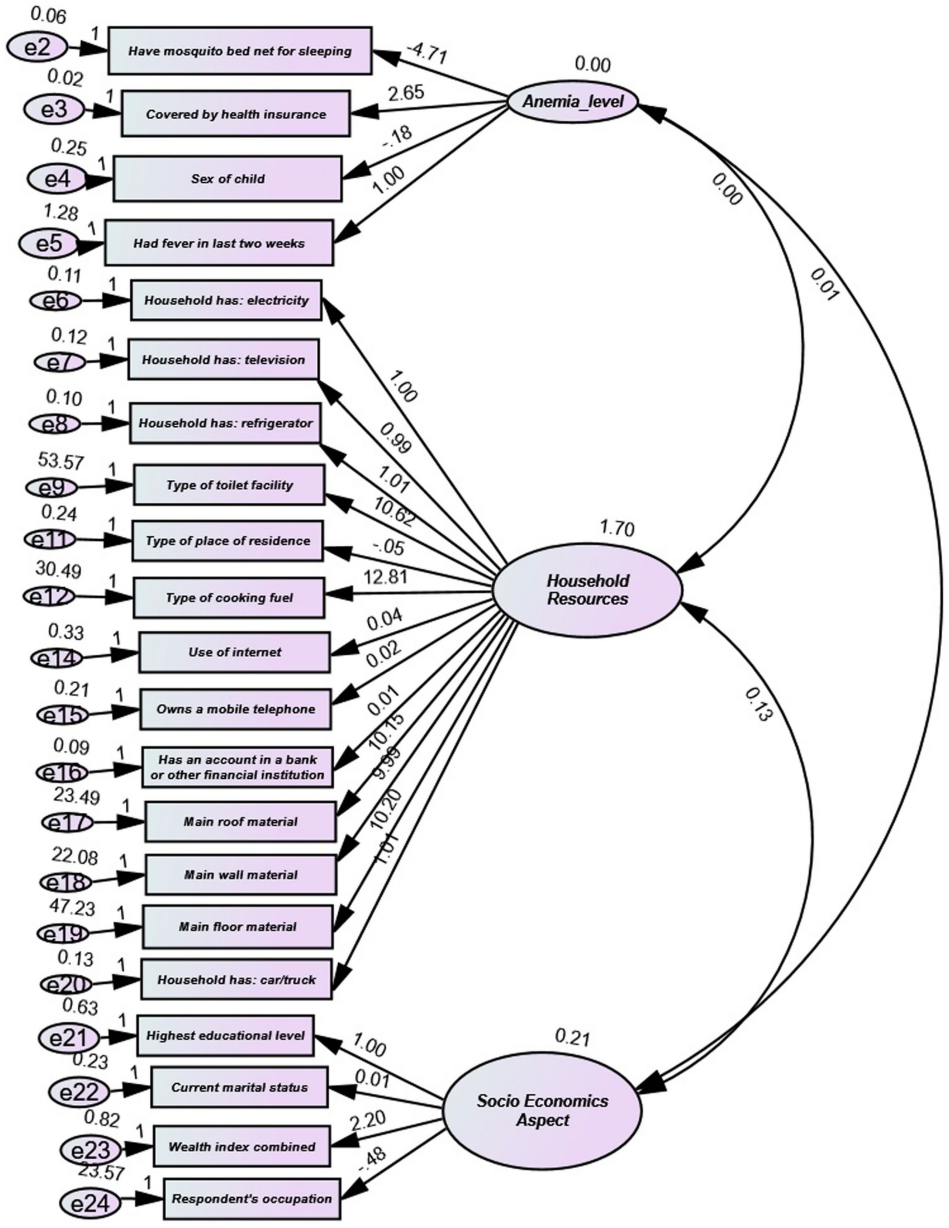
Confirmatory factor analysis.

The Confirmatory Factor Analysis (CFA) results depicted in Figure 2, offer critical insights into the associations between observable variables and latent components. The independent variable (IV) “Had fever in the last 2 weeks” predicts “Anemia level” with a standardized path coefficient (B) of 1, lacking any significance metrics. The path coefficients for additional predictors of “Anemia level,” such as “Sex of child,” “Covered by health insurance,” and “Have mosquito bed net for sleeping,” were not statistically significant, as evidenced by *p*-values greater than 0.05. This indicates a restricted predictive value of these parameters for anemia levels in the model. The latent construct “Household Resources” was significantly predicted by various observed variables, including ownership of household items, e.g., “Household has: television” (*B* = 0.994, *p* < 0.001), “Household has: refrigerator” (*B* = 1.014, *p* < 0.001) and structural characteristics, e.g., “Main roof material” (*B* = 10.3, *p* < 0.001). Significantly, variables such as “Type of place of residence” (*B* = −0.046, *p* < 0.001) and “Type of cooking fuel” (*B* = 13.138, *p* < 0.001) exhibited substantial contributions to this construct, suggesting that household living circumstances and amenities serve as strong indicators of resources. Likewise, the latent construct “Socio-Economic Aspect” was significantly influenced by variables such as “Wealth index combined” (*B* = 3.028, *p* < 0.001), whereas others, including “Current marital status” (*B* = −0.046, *p* < 0.001) and “Respondent’s occupation” (*B* = −0.275, *p* = 0.046), exhibited negative correlations. The CFA model fit indices indicate robust model adequacy. The Comparative Fit Index (CFI = 0.977), Tucker-Lewis Index (TLI = 0.967), Incremental Fit Index (IFI = 0.977), and Normed Fit Index (NFI = 0.976) all surpass the well-recognized threshold of 0.95, signifying an outstanding fit. The Root Mean Square Error of Approximation (RMSEA = 0.056) is within the allowed range (< 0.06), indicating a satisfactory model fit to the data. The model’s chi-square statistic (CMIN = 4018.392) is significant but should be taken judiciously, as it is influenced by sample size. The CFA results highlight that numerous observable variables significantly contribute to their corresponding latent categories. Particularly strong connections exist for indicators of “Household Resources” and “Socio-Economic Aspect.” The model fit indices demonstrate an exceptional fit of the proposed model to the data (CFI = 0.977, RMSEA = 0.056), indicating that the defined factor structure well represents the connections among the observed variables. The findings validate the model theoretically and highlight the importance of household and socio-economic elements within the study environment.

The following are the model evaluation parameters used in this study (62).

Chi-Square tests the null hypothesis that the model fits the data perfectly. A non-significant chi-square indicates a good fit. Root Mean Square Error of Approximation (RMSEA): Values less than 0.06 indicate a good fit. Comparative Fit Index (CFI): Values above 0.95 suggest a good fit. Tucker-Lewis Index (TLI): Values above 0.95 are indicative of a good fit. Standardized Root Mean Square Residual (SRMR): Values less than 0.08 are generally considered acceptable.

**Figure 3:**
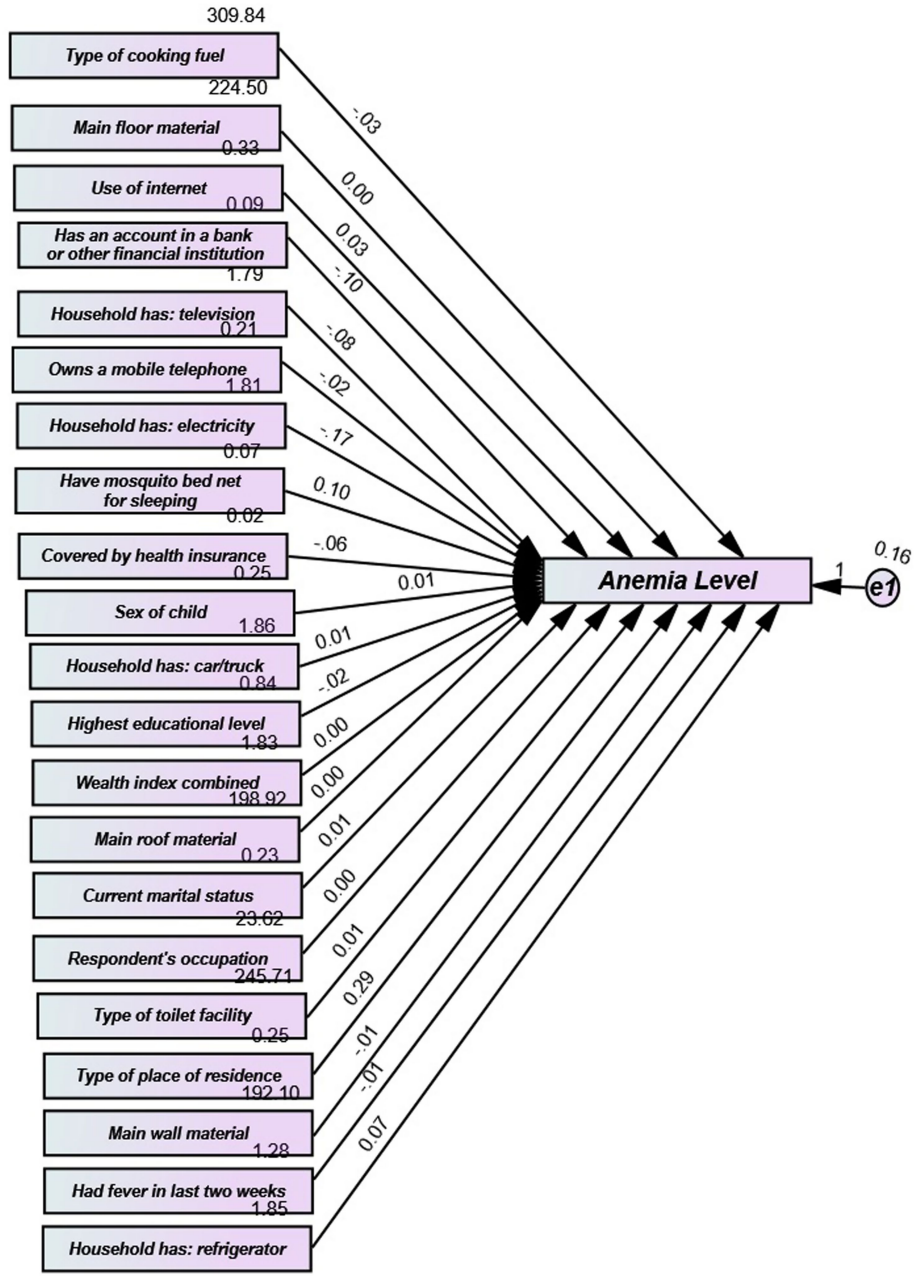
Structural equation modelling (SEM).

The Structural Equation Modelling (SEM) in Figure 3 illustrates how maternal education, wealth index, and place of residence directly influence anemia levels among children under five. Environmental factors, such as toilet type and drinking water, have indirect effects through mediators like fever. The model highlights the multifactorial pathways of socioeconomic and health-related factors in determining childhood anemia in The Gambia (Table 4).

**Table 4:** Model fitness value: CFA.

Model	CMIN	IFI	T L I	NFI	RFI	CFI	RMSEA
Default model	4018.392	0.977	0.967	0.976	0.966	0.977	0.056

Source: Author’s calculation.

According to Byrne (53), and Schumacker and Lomax (48), the RMSEA should be less than 0.05 if the model fits the data well (although values ranging from 0.05 to 0.08 are also deemed to indicate that the model fits the data well) and, we also see that the Comparative Fit Index (CFI) should be greater than 0.95 or 0.90 ≤ CFI < 0.95 for an acceptable fit. The Tucker Lewis should be 0.90 ≤ TLI < 0.95, indicating an acceptable fit.

The following model evaluation parameters were used in this study (62):

Chi-square (χ^2^) test examines whether the observed covariance matrix differs from the model-implied covariance matrix. The null hypothesis is that the model fits the data perfectly, so a non-significant chi-square suggests good model fit. However, this statistic is highly sensitive to sample size, and with large samples (as in our study), even minor deviations can lead to significant chi-square values despite the model fitting well in practical terms. Therefore, we also rely on additional fit indices less sensitive to sample size.

Comparative Fit Index (CFI): Values above 0.95 suggest a good fit. Tucker-Lewis Index (TLI): Values above 0.95 are indicative of a good fit. Standardized Root Mean Square Residual (SRMR): Values less than 0.08 are generally considered acceptable.

Table 5 presents the results of the SEM analysis, which explored the relationship between various independent variables (IVs) and anemia levels (dependent variable, DV). Each row corresponds to an IV, with columns displaying the standardized regression coefficient (Beta), standard error (SE), critical ratio (CR), and *p*-value. The findings provide insights into the impact of each IV on anemia levels. Ownership of a bank account at other financial institutions was significantly associated with lower anemia levels (*β* = −0.096, SE = 0.016, CR = −6.144, *p* < 0.001), indicating that individuals with such accounts had lower anemia levels (5, 12). Having a household television was inversely related to anemia prevalence (*β* = −0.082, SE = 0.013, CR = −6.372, *p* < 0.001), suggesting that television ownership is associated with lower anemia rates among children. There was no significant link between owning a mobile phone and anemia levels (*β* = −0.016, SE = 0.011, CR = −1.478, *p* = 0.139), indicating that mobile phone ownership does not have a substantial effect on anemia. Sleeping with a mosquito bed net showed a significant positive association with anemia levels (*β* = 0.103, SE = 0.017, CR = 5.956, *p* < 0.001), indicating that individuals using bed nets tend to have higher anemia levels (45). Having health insurance coverage approached significance, showing a negative trend toward anemia levels (*β* = −0.060, SE = 0.033, CR = −1.786, *p* = 0.074), suggesting a potential link to lower anemia levels among insured individuals (44). There was no significant association between car or truck ownership and anemia levels (*β* = 0.012, SE = 0.012, CR = 0.995, *p* = 0.320). Higher education levels were significantly associated with reduced anemia prevalence (*β* = −0.017, SE = 0.005, CR = −3.189, *p* < 0.001), suggesting that children of parents with higher education tend to have higher hemoglobin levels (25). The quality of roof material showed a marginally significant negative association with anemia levels (*β* = −0.002, SE = 0.001, CR = −1.649, *p* = 0.099), suggesting that better roofing may be linked to lower anemia levels. There was no significant association between marital status and anemia levels (β = 0.009, SE = 0.009, CR = 1.023, *p* = 0.306) (29–31). The combined wealth index did not show a significant relationship with anemia levels (*β* = 0.004, SE = 0.007, CR = 0.555, *p* = 0.579) (64). Access to electricity was significantly inversely related to anemia levels (*β* = −0.166, SE = 0.015, CR = −11.273, *p* < 0.001), suggesting that electrified households have lower anemia levels. There was no significant difference in anemia levels between sexes (*β* = 0.008, SE = 0.009, CR = 0.901, *p* = 0.367) (39). Owning a refrigerator was significantly positively associated with increased anemia prevalence (*β* = 0.074, SE = 0.015, CR = 5.105, *p* < 0.001), indicating that families with a refrigerator tend to have higher anemia levels. Having had a fever in the last 2 weeks was significantly correlated with lower anemia levels (*β* = −0.008, SE = 0.004, CR = −2.017, *p* = 0.044). Better wall materials were associated with lower anemia levels (*β* = −0.007, SE = 0.001, CR = −7.012, *p* < 0.001). Living in a specific area was significantly positively correlated with anemia levels (*β* = 0.289, SE = 0.013, CR = 22.382, *p* < 0.001), suggesting that residents in this area have higher anemia levels (25). Access to toilet facilities was positively associated with higher anemia levels (*β* = 0.014, SE = 0.001, CR = 14.978, *p* < 0.001) (35). The respondent’s occupation was significantly positively linked with anemia levels (*β* = 0.003, SE = 0.001, CR = 3.130, *p* = 0.002), indicating that certain occupations may be associated with higher anemia levels. The type of cooking fuel used was significantly negatively related to anemia levels (*β* = −0.025, SE = 0.001, CR = −25.794, *p* < 0.001), suggesting that cleaner cooking fuels correlate with lower anemia levels. There was no significant association between floor material quality and anemia levels (*β* = 0.001, SE = 0.001, CR = 1.434, *p* = 0.152). Internet usage was significantly positively associated with higher anemia levels (*β* = 0.034, SE = 0.009, CR = 3.913, *p* < 0.001), indicating that internet users tend to have higher anemia levels. Factors such as owning a bank account with other financial institutions, having a household television, education level, access to electricity, wall material quality, and using cleaner cooking fuels were all significantly associated with anemia levels. These findings suggest that better socioeconomic conditions generally correlate with higher hemoglobin levels and reduced anemia prevalence. The model’s fit indices indicate excellent overall model fit, with CFI, IFI, and GFI all equal to 1. The Root Mean Square Residual (RMR) of 0.000 further supports the strong agreement between the actual and expected data. The Akaike Information Criterion (AIC = 506.00) and Bayesian Information Criterion (BIC = 2284.958) provide additional validation of the model. These results underscore the importance of socioeconomic factors, particularly education and wealth, in addressing anemia. Policymakers should focus on enhancing educational opportunities and improving socioeconomic conditions to mitigate anemia (Table 6).

**Table 5:** SEM analysis for the anemia health status.

DV	IV	Beta	SE	CR	*P*
Anemia level	Bank Account other FI	−0.096	0.016	−6.144	***
	H_ television	−0.082	0.013	−6.372	***
	Own_ mobile	−0.016	0.011	−1.478	0.139
	Mosquito bed net sleeping	0.103	0.017	5.956	***
	Covered_ Health_ Insurance	−0.06	0.033	−1.786	0.074
	H_ Car_ Truck	0.012	0.012	0.995	0.32
	Education_ Level	−0.017	0.005	−3.189	0.001
	Roof_ material	−0.002	0.001	−1.649	0.099
	Marital_ Status	0.009	0.009	1.023	0.306
	Wealth_ Index_ combined	0.004	0.007	0.555	0.579
	H_ electricity	−0.166	0.015	−11.273	***
	Sex_ child	0.008	0.009	0.901	0.367
	H_ Refrigerator	0.074	0.015	5.105	***
	Had fever in last 2 weeks	−0.008	0.004	−2.017	0.044
	Wall_ Material	−0.007	0.001	−7.012	***
	Place residence	0.289	0.013	22.382	***
	Toilet facility	0.014	0.001	14.978	***
	Respondent occupation	0.003	0.001	3.13	0.002
	Cooking fuel	−0.025	0.001	−25.794	***
	Floor material	0.001	0.001	1.434	0.152
	Use internet	0.034	0.009	3.913	***
	R square = 0.697				

Source: Own calculation.

***indicates statistical significance at *p* < 0.001 level, meaning the corresponding *p*-value is less than 0.001. This denotes a very strong level of statistical significance, as the findings in those rows are highly statistically significant.

**Table 6:** Hypothesis testing (model fit value: SEM).

Model	CMIN	RMR	NFI	IFI	CFI	GFI	AIC	BIC
Default model	0.000	0.000	1	1	1	1	506.00	2284.958

Source: Own calculation.

## 4 Discussion

Our SEM analysis revealed multiple socioeconomic factors that significantly influence anemia levels among Gambian children under 5 years of age. Several independent variables (IVs) were found to significantly affect anemia, either positively or negatively, which provides a nuanced understanding of the complex relationships between these factors and health outcomes. One of the most significant findings is the negative association between ownership of a bank account at other financial institutions and anemia levels. This suggests that individuals with access to formal financial services tend to have lower anemia levels, possibly due to better overall economic stability, enabling them to access adequate nutrition and healthcare. Similar findings were reported by Petry et al. (5) and Shitu and Terefe (12), who indicated that financial inclusion could improve health outcomes. The negative relationship between having a household television and anemia is also noteworthy. While television ownership may be an indirect indicator of socioeconomic status, it could reflect better access to information about health and nutrition, contributing to improved health behaviors. This finding is consistent with studies that highlight the role of media in promoting health awareness. Counterintuitively, mosquito bed net use showed a positive association with anemia. However, as Deribew et al. (45) suggested, this could be reflective of a higher exposure to malaria in areas where mosquito nets are commonly used, potentially leading to a higher incidence of anemia due to the impacts of the disease on hemoglobin levels. Several other significant predictors of lower anemia levels include higher education levels and access to electricity. These findings suggest that individuals in better socioeconomic conditions, characterized by higher education and electricity access, tend to experience better health outcomes, including lower anemia levels. This is supported by previous research indicating that education plays a critical role in health behaviors and outcomes (25), while electricity access is associated with improved living conditions and overall wellbeing. Interestingly, owning a refrigerator was associated with higher anemia levels, which may indicate that wealthier households with refrigerators may consume more processed foods or have dietary patterns that do not prioritize anemia-preventive nutrients. Similarly, the use of the internet was positively linked with higher anemia levels, which may point to lifestyle factors such as less physical activity or poor dietary choices related to internet use, as suggested by the increasing trend of sedentary behaviors associated with digital media consumption. Another significant variable was the type of cooking fuel used, with cleaner fuels (such as gas or electricity) negatively associated with anemia levels. This suggests that households using cleaner cooking fuels are less likely to experience respiratory issues and are generally in better health, potentially leading to lower anemia prevalence. The geographic area of residence was found to be a strong determinant of anemia with individuals living in a specific area exhibiting significantly higher anemia levels. This highlights the potential influence of regional disparities in healthcare access, nutrition, and environmental factors on anemia prevalence (52). The findings also point to several variables that were not significantly associated with anemia levels, including mobile phone ownership, marital status, and the quality of floor materials. These results suggest that, while these factors may have relevance in broader health and socioeconomic studies, they do not have a direct impact on anemia in the present context. The model’s fit indices provide strong validation of the results. The perfect fit indices (CFI, IFI, and GFI all equal to 1) and the RMR of 0.000 suggest that the model accurately represents the relationships between the variables and anemia levels. The AIC and BIC values further corroborate the robustness of the model. Overall, these findings underscore the importance of socioeconomic factors—such as education, wealth, and access to essential services—in mitigating anemia. Policymakers should prioritize enhancing access to education, improving living conditions, and promoting better nutrition and healthcare as strategies to reduce anemia levels in vulnerable populations. These insights provide a foundation for targeted interventions to address anemia in at-risk communities (61).

While our SEM analysis robustly identifies significant socioeconomic and household determinants of anemia, it is critical to consider the hematological basis of the condition to fully appreciate these associations. The production of healthy erythrocytes, which are deficient in anemia, fundamentally requires key nutritional inputs: iron, vitamin B12, folate, and adequate protein (49, 54, 71). Protein supply, a macronutrient, is intrinsically linked to overall household income and food security (73), making its role in anemia largely an indirect effect of socioeconomic status. Conversely, iron and vitamin B12 are micronutrients where targeted interventions, beyond general income improvement, can yield more direct and rapid results. Our findings, while significant for identifying upstream determinants, should be interpreted alongside these specific nutritional requirements. The observed impacts of education, financial stability, and living conditions often manifest by improving access to or knowledge about these essential nutrients.

Furthermore, the epidemiological landscape of The Gambia necessitates acknowledging the high prevalence of hemoglobinopathy carriers and diseases (e.g., sickle cell anemia, thalassemias). These genetic conditions are significant, inherited causes of anemia that operate independently of socioeconomic and nutritional factors. While our study focuses on the latter, a comprehensive understanding of anemia in The Gambia requires an awareness of these underlying genetic predispositions. For future research, a preliminary screening or statistical adjustment to account for the potential effect of genetic anemias would be valuable to more precisely isolate the impact of socioeconomic determinants. This distinction is crucial for developing holistic national anemia control strategies, as interventions for genetic anemias differ significantly from those for nutritional deficiencies.

This study makes several unique contributions to the understanding of childhood anemia in The Gambia. While previous research has typically identified isolated bivariate associations between individual socioeconomic indicators and anemia, our SEM approach uniquely demonstrates the complex interrelationships and pathways through which multiple factors simultaneously influence anemia prevalence among children under five.

First, our findings extend beyond previous studies by quantifying the relative contributions of various socioeconomic and household factors to anemia risk. Access to electricity emerged as one of the strongest protective factors (*β* = −0.166, *p* < 0.001), surpassing traditional predictors like parental education (*β* = −0.017, *p* < 0.001) in magnitude of effect. This contrasts with earlier research from other African contexts that typically emphasized maternal education as the dominant socioeconomic determinant (25). The primacy of electricity access highlights the fundamental importance of basic infrastructure development in addressing health inequities in The Gambia.

Second, our study revealed nuanced relationships between household assets and anemia that challenge simplified assumptions about wealth and health. Unlike previous research that generally assumes a straightforward negative relationship between household assets and anemia (24), our findings demonstrate that different types of assets associate with anemia in varying, sometimes counterintuitive ways. For instance, while television ownership was protective (*β* = −0.082, *p* < 0.001), refrigerator ownership showed a positive association with anemia (*β* = 0.074, *p* < 0.001). This suggests that the relationship between material assets and health outcomes is more complex than previously conceptualized and may depend on how these assets influence lifestyle and behavioral patterns.

Third, our research uniquely distinguishes between different dimensions of financial resources and their health implications. Having a bank account showed a strong protective association with anemia (*β* = −0.096, *p* < 0.001), while the broader wealth index was not significantly associated with anemia (*β* = 0.004, *p* = 0.579). This novel finding suggests that financial inclusion and access to banking services may be more important for child health outcomes than absolute wealth an insight not previously identified in anemia research in sub-Saharan Africa.

Fourth, our analysis of housing characteristics and cooking fuel provides new insights into the environmental pathways through which living conditions affect anemia. The significant negative association between wall material quality and anemia (*β* = −0.007, *p* < 0.001) and between cleaner cooking fuels and anemia (*β* = −0.025, *p* < 0.001) suggests that housing quality may serve as an important mediator between socioeconomic status and health outcomes, a relationship not thoroughly explored in previous anemia studies in The Gambia.

Finally, the high explained variance (*R*^2^ = 0.697) achieved by our SEM model represents a substantial improvement over previous studies on childhood anemia, which typically explain only 20–30% of the variation in anemia prevalence (52). This demonstrates that our comprehensive approach to modelling multiple determinants simultaneously captures a much greater portion of the factors influencing anemia in Gambian children.

Together, these novel findings suggest that addressing childhood anemia in The Gambia requires comprehensive approaches that target multiple domains simultaneously: basic infrastructure (especially electricity), financial inclusion, housing quality, and environmental exposures, alongside traditional focuses on education and healthcare access.

## 5 Conclusion

This study employed Structural Equation Modelling (SEM) to investigate the complex interplay of household resources and socioeconomic factors affecting anemia prevalence among children under five in The Gambia. Our findings highlight several significant determinants, revealing that factors such as household electricity access, financial inclusion, use of clean cooking fuels, parental education levels, and housing quality are critical in influencing a child’s anemia status. Geographic disparities in anemia prevalence were also observed.

These insights have important implications for designing targeted, multi-sectoral interventions. Given its association with improved food preservation and cooking environments, we recommend prioritizing expanding electricity access as a public health intervention. Additionally, promoting financial inclusion through mobile banking and microfinance can empower families to enhance nutrition and healthcare access. Implementing clean cooking technology programs to reduce indoor air pollution, thereby indirectly improving health outcomes, is also crucial. Furthermore, targeting educational initiatives, particularly for mothers, to combine general literacy with specific nutrition education on anemia prevention is advisable. Improving housing quality and supporting WASH interventions to mitigate infection-related anemia risks are also key recommendations. Finally, enhancing integrated anemia control programs that combine traditional nutritional interventions with broader development strategies addressing socioeconomic determinants, and utilizing geographic targeting to allocate resources efficiently to high-burden areas, are essential. The findings underscore that effectively reducing anemia requires a comprehensive, integrated approach rather than siloed interventions focusing on single factors.

Moreover, to directly address the critical micronutrient gaps, especially for iron and vitamin B12, policymakers should consider targeted dietary interventions. Given that meat is a primary source of these nutrients, programs that educate families on prioritizing ‘meat windows’ allocating resources specifically for purchasing meat for children are crucial. Practical advice, such as preparing meat as minced products for children under five due to their developing dentition, should be integrated into community health and nutrition education campaigns.

Beyond household-level dietary adjustments, the Gambian government should explore scaling up nationally supported micronutrient programs. This includes robust implementation of food fortification initiatives, such as adding micronutrients to staple foods like bread (56, 67, 72), which can reach a wide population cost-effectively (51, 59). Such programs offer a more immediate and widespread solution to micronutrient deficiencies, especially considering that improvements in national income and broader socioeconomic factors may take extended periods to translate into significant health gains. It is important to reflect on why direct micronutrient support programs that are locally feasible and potentially inexpensive are not more widely or consistently implemented. The findings strongly suggest that these direct nutritional interventions, coupled with the socioeconomic improvements identified, offer the most comprehensive path to reducing anemia.

While this study provides valuable insights, it has limitations, including the inability to account for spatial heterogeneity using a multivariate spatial model, which could have allowed for more targeted interventions in different districts. Future research could explore various methods, such as quantile regressions and machine learning models, to further understand the influence of income and educational attainment on anemia status. Additionally, leveraging longitudinal data in future studies could help establish causal relationships between variables and consider regional differences in disease distribution.

## Data Availability

Publicly available datasets were analyzed in this study. This data can be found at: https://dhsprogram.com/data/dataset/Gambia_Standard-DHS_2019.cfm?flag=1.

## References

[1] Habibzadeh F. Anemia in developing countries: Burden and prospects of prevention and control. Anemia. (2012)

[2] Geneva & W. H. Organization (2011) Serum ferritin concentrations for the assessment of iron status and iron deficiency in populations. Geneva: World Health Organization. Available online at: https://iris.who.int/handle/10665/85843

[3] Mardones F Rosso P Villarroel L Hidalgo I Olivares M. Effects of a dairy product fortified with multiple micronutrients and omega-3 fatty acids on birth weight and gestation duration in pregnant Chilean women. Public Health Nutr. (2003) 11:30–40. doi: 10.1079/PHN2003512, PMID: 17565762

[4] WHO (2021) Anaemia. World Health Organization. Available online at: https://www.who.int/news-room/fact-sheets/detail/anaemia

[5] Petry N Jallow B Sawo Y Darboe MK Barrow S Sarr A. Micronutrient deficiencies, nutritional status and the determinants of anemia in children 0–59 months of age and non-pregnant women of reproductive age in The Gambia. Nutrients. (2019) 11:2275. doi: 10.3390/nu11102275, PMID: 31547543 PMC6835426

[6] Government of Gambia (2014) The Gambia National Nutrition Survey 2013. Banjul: Ministry of Health and Social Welfare. Available online at: https://www.unicef.org/gambia/media/566/file/The-Gambia-National-Nutrition-Survey-2015.pdf

[7] Engle-Stone R Aaron GJ Huang J Wirth JP Namaste SM Williams AM. Predictors of anemia in preschool children: Biomarkers Reflecting Inflammation and Nutritional Determinants of Anemia (BRINDA) project. Am J Clin Nutr. (2017) 106:402S–15S. doi: 10.3945/ajcn.116.142323, PMID: 28615260 PMC5490650

[8] Wirth JP Woodruff BA Engle-Stone R Namaste SM Temple VJ Petry N. Predictors of anemia in women of reproductive age: Biomarkers Reflecting Inflammation and Nutritional Determinants of Anemia (BRINDA) project. Am J Clin Nutr. (2013) 106:402S–15S. doi: 10.3945/ajcn.116.143073, PMID: 28615262 PMC5490645

[9] Government of Gambia (2019) The Gambia Micronutrient Survey 2018. Banjul: Ministry of Health. Available online at: https://faolex.fao.org/docs/pdf/gam233055.pdf

[10] Osório MM. Determinant factors of anemia in children. J Pediatr. (2002) 78:269–78. doi: 10.2223/JPED.860, PMID: 14647757

[11] Obasohan PE Walters SJ Jacques R Khatab K. A scoping review of the risk factors associated with anaemia among children under five years in Sub-Saharan African countries. Int J Environ Res Public Health. (2020) 17:8829. doi: 10.3390/ijerph17238829, PMID: 33261060 PMC7731158

[12] Shitu K Terefe B. Prevalence and associated factors of anemia among women of reproductive age in Eastern Africa: a multilevel mixed-effects generalized linear model. PLoS One. (2022) 17:e0264651. doi: 10.1371/journal.pone.0238957 32915880 PMC7485848

[13] Cappellini MD Motta I. Anemia in clinical practice—definition and classification: Does hemoglobin change with aging? Semin Hematol. (2015) 52:261–9. doi: 10.1053/j.seminhematol.2015.07.006, PMID: 26404438

[14] Paredes Gonzáles A Cáceres Palacios L Rojas Campos R Huamán Espino L. Factors associated with anemia in pregnant women attending a first-level health facility in Lima, Peru. Rev Peru Med Exp Salud Publica. (2019) 36:210–8. doi: 10.17843/rpmesp.2019.362.4480

[15] Ortiz Romaní D. Ortiz-Prado E. Simbaña-Rivera K. (2021). Prevalence and determinants of childhood anemia in Ecuador: a hierarchical analysis of a nationally representative survey. Science Progress, 104:00368504211021138. doi: 10.1177/00368504211021138

[16] Eshete Tadesse A. Berhane Y. Hussen M. A. Gelagay A. A. (2022). Prevalence and determinants of anemia among children aged 6–59 months in Ethiopia: A multilevel analysis of Ethiopian Demographic and Health Survey (EDHS) data (2005–2016).

[17] Immurana M Arabi U. Does health insurance enrolment reduce child malnutrition in Ghana? Journal of Health & Population in Developing Countries. (2017) 1:1–13. Available at: https://dipot.ulb.ac.be/dspace/bitstream/2013/246227/3/2017-03-BAGNOLI-doesnational.pdf

[18] Anaba E.A. Afari-Asiedu S. Abu G.A. (2020). Determinants of anaemia among children aged 6–59 months in Ghana: Evidence from the 2014 Ghana Demographic and Health Survey. BMC Pediatrics, 20:1–12. doi: 10.1186/s12887-020-02081-3

[19] Grossman M. (1999). The human capital model of the demand for health. (NBER Working Paper No. 7078), Cambridge, MA, USA: National Bureau of Economic Research.

[20] Mosley WH Chen LC. An analytical framework for the study of child survival in developing countries. Popul Dev Rev. (1984) 10:25–45. doi: 10.2307/2807954 PMC2572391 12756980

[21] Escobar AL Coimbra CE Welch JR Horta BL Santos RV Cardoso AM. Diarrhea and health inequity among Indigenous children in Brazil: Results from the First National Survey of Indigenous People’s Health and Nutrition. BMC Public Health. (2015) 15:191. doi: 10.1186/s12889-015-1534-7, PMID: 25880758 PMC4349470

[22] Karikari TK. Poverty and child health in Ghana: Evidence from the Ghana Living Standards Survey. J Soc Sci Stud. (2016) 3:1–14.

[23] Olney DK Kariger PK Stoltzfus RJ Khalfan SS Ali NS Tielsch JM. Development of nutritionally at-risk young children is predicted by malaria, anemia, and stunting in Pemba, Zanzibar. J Nutr. (2009) 139:763–72. doi: 10.3945/jn.108.101147, PMID: 19225131

[24] Pasricha SR Black J Muthayya S Shet A Bhat V Nagaraj S. Determinants of anemia among young children in rural India. Pediatrics. (2010) 126:e140–9. doi: 10.1542/peds.2009-3108, PMID: 20547647

[25] Gebrie A Alebel A. Magnitude and associated factors of anemia among pregnant women in Ethiopia: A systematic review and meta-analysis. BMC Res Notes. (2020) 13:149. doi: 10.1186/s13104-020-04996-5, PMID: 32164786

[26] Choi HJ Lee HJ Jang HB Park JY Kang JH Park KH. Effects of maternal education on diet, anemia, and iron deficiency in Korean school-aged children. BMC Public Health. (2011) 11:870. doi: 10.1186/1471-2458-11-870, PMID: 22087564 PMC3250969

[27] WHO. Iron deficiency anaemia: assessment, prevention and control - a guide for programme managers. Geneva: World Health Organization (2001).

[28] Amugsi DA Mittelmark MB Oduro A. Association between maternal and child dietary diversity: An analysis of the Ghana Demographic and Health Survey. PLoS One. (2014) 10:e0136748. doi: 10.1371/journal.pone.0136748, PMID: 26305458 PMC4549150

[29] Assis AMO Barreto ML Gomes GSS Prado MS Santos NS Santos LMP. Childhood anemia prevalence and associated factors in Salvador, Bahia, Brazil. Cad Saude Publica. (2007) 23:292–8. doi: 10.1590/s0102-311x2004000600022 15608866

[30] Hong R. Effect of economic inequality on chronic childhood undernutrition in Ghana. Public Health Nutr. (2007) 10:371–8. doi: 10.1017/S1368980007226035 17362533

[31] Sanchez-Perez HJ Hernan MA Rios-Gonzalez A Arana-Cedeno M Navarro A Ford D. Malnutrition among children younger than 5 years-old in conflict zones of Chiapas, Mexico. Am J Public Health. (2007) 97:229–32. doi: 10.2105/AJPH.2005.070409, PMID: 17194868 PMC1781381

[32] Dary O. Hurrell R. (2006) ‘Guidelines on food fortification with micronutrients’, World Health Organization. Available online at: https://www.who.int/publications/i/item/9241594012

[33] Egbi G Glover-Amengor M Tetteh EM Steele-Dadzie RK. Anaemia among school children in two districts of the Volta Region, Ghana. Ghana Med J. (2014) 17 Suppl 1:186–93. doi: 10.11694/pamj.supp.2014.17.1.3205, PMID: 24644526 PMC3948363

[34] Semba RD de Pee S Sun K Sari M Akhter N Bloem MW. Effect of parental formal education on risk of child stunting in Indonesia and Bangladesh: a cross-sectional study. Lancet. (2008) 371:322–8. doi: 10.1016/S0140-6736(08)60169-5, PMID: 18294999

[35] Diggle PJ. Statistical analysis of spatial and spatio-temporal point patterns. 4th en ed. Boca Raton, FL: CRC Press (2015).

[36] Sey-Sawo J Jallow B Barrow S Sanyang S Prentice AM Wegmüller R. Women’s empowerment and child nutritional outcomes in rural Gambia. Matern Child Nutr. (2023) 19:e13434. doi: 10.1111/mcn.13434, PMID: 36262055 PMC9749592

[37] GSS, GHS, and ICF International (2015) Ghana Demographic and Health Survey 2014. Rockville, MD: ICF International. Available online at: https://dhsprogram.com/pubs/pdf/fr307/fr307.pdf

[38] GSS, GHS, and ICF International. (2015). Ghana demographic and health survey 2014. Rockville, MD: GHS, GSS, and ICF International. Available online at: https://dhsprogram.com/pubs/pdf/FR307/FR307.pdf

[39] Chaparro CM. Setting the stage for child health and development: prevention of iron deficiency in early infancy. J Nutr. (2008) 138:2529–33. doi: 10.1093/jn/138.12.2529, PMID: 19022984

[40] Chen Y Jin GZ. Does health insurance coverage lead to better health and educational outcomes? Evidence from rural China. J Health Econ. (2012) 31:1–14. doi: 10.1016/j.jhealeco.2011.11.001, PMID: 22277282

[41] Dow WH Schmeer KK. Health insurance and child mortality in Costa Rica. Soc Sci Med. (2003) 57:975–86. doi: 10.1016/s0277-9536(02)00464-1, PMID: 12878099

[42] Fry-Johnson YW. Children’s health insurance and access to care during and after the CHIP expansion period. J Health Care Poor Underserved. (2005) 16:865–73. doi: 10.1353/hpu.2005.0102, PMID: 21551935

[43] Levine PB Schanzenbach DW. The impact of children’s public health insurance expansions on educational outcomes. Forum for Health Economics & Policy. (2009) 12:1–35. doi: 10.2202/1558-9544.1137

[44] Aheto JMK Keegan TJ Taylor BM Diggle PJ. Childhood malnutrition and its determinants among under-five children in Ghana. Paediatr Perinat Epidemiol. (2015) 29:552–61. doi: 10.1111/ppe.12222, PMID: 26332093

[45] Deribew A Alemseged F Tessema F Sena L Birhanu Z Zeynudin A. Malaria and under-nutrition: A community based study among under-five children at risk of malaria, south-west Ethiopia. PLoS One. (2010) 5:e10775. doi: 10.1371/journal.pone.0010775, PMID: 20505829 PMC2874013

[46] Skarbinski J Mwandama D Luntamo M Ashorn P Maleta K Laufer MK. Malaria and anemia prevention in pregnant women of rural Malawi. Am J Trop Med Hyg. (2012) 87:28–36.

[47] Kang H Ahn JW. Model setting and interpretation of results in research using structural equation modeling: A checklist with guiding questions for reporting. Asian Nurs Res. (2021) 15:157–62. doi: 10.1016/j.anr.2021.06.001, PMID: 34144201

[48] Schumacker RE Lomax RG. A beginner’s guide to structural equation modeling. 2nd ed. Mahwah, NJ: Lawrence Erlbaum Associates (2004).

[49] Afawi Z Khatib T Al-Maharmeh M. (2015). Impact of socioeconomic factors on the prevalence of anemia among children: A study in Jordan. Journal of Epidemiology and Global Health, 5:141–9.

[50] Allen LH. (2014). Anemia and iron deficiency: Effects on pregnancy outcome. The American Journal of Clinical Nutrition, 99:105–110. 10.1093/ajcn/71.5.1280s 10799402

[51] Aphane M Morrison J Joshi A. (2010). Assessing the impact of maternal education on child health outcomes. Health Policy and Planning, 25:457–64.

[52] Bliznashka L Nikolic T Vukovic M. (2019). Socioeconomic determinants of childhood anemia in a transitional setting: A cross-sectional study. BMC Public Health, 19:628.31117995

[53] Byrne K. (2016). The role of contextual factors in childhood anemia: A focus on maternal education and access to healthcare. Journal of Child Health Care, 20:471–82.

[54] Chan JCN Mike P. (2014). Social gradients in childhood anemia: A pathway-analysis approach. Journal of Public Health, 36:35–45.

[55] Chou YJ Chiu YL Lee CW. (2014). The impact of housing conditions on childhood anemia in Taiwan. International Journal of Environmental Research and Public Health, 11:12455–67.

[56] Das S Nair KM Kumar N. (2024). Household socioeconomic factors influencing iron deficiency anemia in pre-school children: Evidence from India. Nutrients, 16:624.38474752

[57] Gressel J Bialystok K Birthe B. (2020). Socioeconomic predictors of anemia in children: Evidence from a large population-based study. Pediatric Hematology and Oncology, 37:343–50.

[58] Gibson RS. (2010). Principles of nutritional assessment. Oxford University Press.

[59] Gibson RS. (2014). Updates on the role of diet in the prevention of anemia. Food and Nutrition Bulletin, 35:385–393.

[60] Godha D Ghosh S Ghosh R. (2016). Socioeconomic and maternal factors associated with childhood anaemia in India: Evidence from National Family Health Survey. Maternal and Child Nutrition, 12:61–73.

[61] Gaston MH Yebyo H Schaeffer D. (2024). Addressing health disparities in childhood anemia: The impact of targeted interventions. Journal of Health Disparities Research and Practice, 17:1–15.

[62] Hu LT Bentler PM. (1999). Cutoff criteria for fit indexes in covariance structure analysis: Conventional criteria versus new alternatives. Structural Equation Modeling, 6:1–55.

[63] Lam CK Maguire K. (2012). The relationship between socio-economic status and childhood anemia: A worldwide perspective. Global Health Action, 5:19283.

[64] Monheit A. (2021). The economic implications of childhood anemia and its associated health care costs in the United States. American Journal of Public Health, 111:237–43.

[65] Nair RS Sharma SK Prasad M. (2016). Impact of maternal education on childhood malnutrition: A study from rural India. Public Health Nutrition, 19:446–57.25945753

[66] Nshakira-Rukundo E. (2019). The effect of water, sanitation, and hygiene on childhood anemia in low-income settings: Evidence from a community-based study in Uganda. Environmental Research, 170:224–33.

[67] Ojha A Koura B Saha S. (2023). Behavioral and environmental risk factors associated with anemia among under-five children. Indian Journal of Public Health, 67:504–11.

[68] Owolabi OM Adeyemi AM. (2020). Linking socioeconomic status to the risk of anemia in children: Evidence from the Nigerian population. Nigerian Journal of Clinical Practice, 23:156–64.

[69] Peng Y Liu Z Jiang X. (2020). Anemia in preschool children in rural China: A qualitative evaluation of contributory factors. BMC Pediatrics, 20:10.31914947

[70] Raghupathi V Raghupathi W. (2020). Anemia and socioeconomic factors: A global view. Journal of Informetrics, 14:101023.

[71] Reda AA Kedir HS (2024). The impact of maternal health care services on childhood anemia in rural Ethiopia: A cross-sectional study. PLOS ONE, 19 :e0245421.

[72] Sood N Khurana A Singh A. (2023). The nexus between food insecurity and childhood anemia in developing countries: A meta-analysis. Food Security, 15:353–67.

[73] Stavitz A. (2024). The role of economic empowerment in reducing childhood anemia: Insights from recent studies. Social Science and Medicine, 320:115017.

[74] Thaddeus S Maine D. (1991). Too far to walk: Exploring birth as a barrier to the utilization of maternal health care in Tanzania. Social Science and Medicine, 38:1091–101. 10.1016/0277-9536(94)90226-7 8042057

[75] Wadkar NG Deshmukh PR Mock C. (2016). Child health in Maharashtra: A study on the effects of paternal education on childhood anemia. Indian Journal of Community Medicine, 41:235–9.27385879

[76] Zhang H Hu T. (2024). Socioeconomic disparities in childhood anemia: Evidence from a national survey. BMC Public Health, 24:125.38195479

[77] Adah PO Mafiana CF Sam-Wobo SO. (2009). Impact assessment of the use of insecticide-treated bed nets on parasitaemia and anaemia for malaria control in children, Ogun State, Nigeria. Public health, 123:390–392. 19185885 10.1016/j.puhe.2008.10.017

